# Determination of age reference standards based on mandibular third molar root development in a Ugandan population aged 10–22 years

**DOI:** 10.1186/s41935-022-00308-z

**Published:** 2022-12-07

**Authors:** Annet Kutesa Mutebi, Adriane Kamulegeya, Grace Nabaggala, Cathy Lutalo Mwesigwa

**Affiliations:** Department of Dentistry, College of Health Sciences, Makerere University, P.O. Box 7072, Kampala, Uganda

**Keywords:** Third molar, Age estimation, Root development, Left mandibular molar

## Abstract

**Background:**

This study was aimed at establishing age estimates based on distal root development of the mandibular third molar for Ugandans aged 10–22 years. This was a cross-sectional study using orthopantomograms (OPGs) of 671 patients attending the Mulago Hospital Dental Clinic. The patients’ chronological age and sex were obtained from either their national identity cards or birth certificates (females; *n* = 326, 48.6%). Third molar root development was assessed using Demirjian, Goldstein, and Tanner (DGT), modified Demirjian by Solari, Moorrees, Fanning, and Hunt (MFH), and Haavikko methods. Age was summarized using means/standard deviation (SD), medians, and lower and upper quartiles. Sex differences were assessed using Student’s *t*-test.

**Results:**

Crown completion (stage D) and root initiation (stage R_i_) were observed at 12.6 years for females and 13.5 years for males (*P* = 0.02), while complete apex closure occurred at 19.8 for females and 20.1 for males (*P* = 0.3). There were statistically significant differences in Demirjian root stages E, F, and G and MFH and Haavikko stages R_i_, R_1/4_, and R_1/2_ between the sexes (*P* < 0.05). The difference in the mean age of root development between females and males ranged between 0.9 year at DGT/MFH root stages D and R_i_ (13.5–12.6) and 1.4 years at Solari, MFH, and Haavikko root stages F, R_1/4_, and R_1/2_ (16.3–14.9, 17.1–15.7). No differences were observed between the right and left mandibular molars.

**Conclusions:**

This study provides age reference standards based on third molar root development specific to the Ugandan adolescent and young adult population. The findings can be used to formulate contemporary standards and utilized as reference material to assess third molar maturity for forensic purposes.

## Background

Tooth development is a biological marker with sequential phases of tooth eruption, exfoliation, and maturation, making it suitable for use in age estimation (Kjaer, 2014). This method is particularly reliable during early adolescence (10–14 years of age), the time when several teeth are undergoing development. However, challenges arise during late adolescence when all the permanent teeth have fully developed apart from the third molar (Acharya AB, 2010). Age estimation of individuals aged 15 years and above can rely on the third molar whose development falls within 15- to 25-year age range (Schmeling, Garamendi, Prieto, & Landa, 2011). The third molar is a tooth of interest in forensic and legal practice as a tool of age estimation in late adolescence (Schmeling et al., 2011).

In the absence of valid identification documents, precise age estimation of persons in Uganda is usually a requirement under the law in order to establish whether a person is a juvenile or an adult. This information is then used to determine whether the criminal law for adults or juveniles can be applied. This information is then used to determine whether the criminal law for adults or juveniles can be applied. At 12 years, a person is considered to be criminally irresponsible, while 18 years old is the age of an adult person. The commonly used method to establish the age of juveniles undergoing judicial proceedings in Uganda is third molar eruption.

However, the third molar is reported to have a high level of variability regarding its size, shape, timing of formation, and eruption (Garn, Lewis, & Bonné, 1962a; Levesque, Demirjian, & Tanguay, 1981) and thus anatomically unsuitable for age estimation. As an alternative to tooth eruption, other methods based on third molar development have been developed by different authors (Moorrees, Fanning, & Hunt Jr, 1963; Olze et al., 2006; Thevissen, Fieuws, & Willems, 2010). However, Demirjian, Goldstein, and Tanner (1973) method is the most popular because of the clearly defined stages of tooth development and fewer intermediate stages allowing for better reproducibility (Dhanjal, Bhardwaj, & Liversidge, 2006).

Although the Demirjian, Goldstein, and Tanner (1973) method has previously been used in the Ugandan population (Mwesigwa, Kutesa, Munabi, Kabenge, & Buwembo, 2019), it was based on limited root development stages. The present study considered three additional methods with finer grading of the root development stages in order to establish age estimate standards in relation to the distal root development of the third mandibular molar among Ugandans aged 10–22 years.

## Methods

### Study design and setting

This was a cross-sectional study based on orthopantomographs (OPGs) and interviews of patients attending the dental clinic in Mulago Hospital. The hospital is the national referral and teaching facility located in the capital city, Kampala, with a bed capacity of 1500. Review of records indicated that the dental clinic attends to between 1500 and 2000 patients per month, with approximately 50% aged 10–20 years. These patients are routinely attended to by a team of oral and maxillofacial surgeons, dental surgeons, intern dentists, and dental nurses.

### Study population and participant selection

The study population constituted indigenous Ugandan patients aged 10–22 years attending the dental clinic between January and December 2017. The sample size (*n* = 1089) was calculated using the formulae for sample size determination for a mean in one group. To cater for the comparisons based on sex, equal proportions were selected for the study. All eligible participants who consented were consecutively recruited based on their date of birth, which was used to calculate the respective age. The national identity card was used to ascertain the age of participants in the 16–22 age range, while a birth certificate was used for those aged 10–15 years. In instances where the patients did not have the required documentation, they were requested to return on a subsequent appointment with the documents before recruitment into the study. For cases where the recruited patients did not return, we continued sampling until the sufficient sample size was achieved. Patients with missing mandibular third molars were excluded from the study.

### Data collection procedure

The demographic information of the participants such as sex and date of birth was recorded onto a form. They were coded using identification numbers, which created confidentiality and blinding to the examiners. The radiographic images of the participants were taken under routine care at the dental clinic by an experienced radiographer using a digital orthopantomograph (OPG) machine (Carestream Co., France, 2014). The images were uploaded onto a computer and interpreted by 2 trained and calibrated dentists (AKM and CML). Radiographic images (*n* = 31) with missing mandibular third molars and those of poor quality (blurred images) were excluded from the study. The calibration of the 2 dentists was done through an initial training session in order to harmonize the process of radiographic interpretation. The stage of root development was recorded based on 4 criteria previously described (Table 1), and in instances of questionable or borderline cases, inter-examiner consultation was done in order to minimize variability of the recordings.

**Table 1:** Description of the criteria for recording root development stages of the mandibular third molar

Demirjian stages of calcification	Haavikko stages of calcification	Solari stages of calcification	Moorrees stages of calcification	Stage terminology	Description of stage according to image
D	R_i_	D	R_i_	Root initial/crown complete	Some root visible but less than half crown height
E	R_1/4_	E	R_cl_	Cleft	Beginning of root furcation visible as a dot or line
			R_1/4_	Root one-quarter	Clear semilunar furcation visible
F	R_1/2_	F	R_1/2_	Root one-half	Root bifurcation more extensive. Root length equal to crown height. Distal root canal walls diverge with sharp edges
	R_3/4_	F_1_	R_3/4_	Root three-quarters	Root length considerably more than crown height and root canal walls diverge
G	R_c_	G	R_c_	Root complete	Walls of the distal root canal are parallel and full length with rounded/blunt edges
		G_1_	A_1/2_	Apex half closed	Apex of distal root partially open. Periodontal ligament slightly wider at distal apex
H	A_c_	H	A_c_	Apex closed	Distal apex appears closed with uniform periodontal ligament width

Adapted from Liversidge and Marsden (2010)

### Dental development assessment

The developmental stages of the distal root of the left and right mandibular third molars were considered for this study. This was because the image of the maxillary third molar is often superimposed over other anatomic structures, thus presenting with a distorted image on radiograph. The radiographic images were assessed using four methods of dental development as illustrated in Table 1; the Demirjian et al. (Demirjian, Goldstein, & Tanner, 1973) method employed 4 stages of root development. This method in addition to three other methods is as follows:

- Solari and Abramovitch (Solari & Abramovitch, 2001) which is modified Demirjian
- Haavikko (Haavikko, 1970) and Moorrees et al. (Moorrees et al., 1963) were adopted for this present study.
- Solari and Abramovitch modified (Solari & Abramovitch, 2001) the Demirjian method by employing 7 stages of root development through splitting stages F and G, while Haavikko (Haavikko, 1970) utilized 6 stages of root development by splitting Demirjian stage F.
- The Moorrees method utilized 8 stages of root development by splitting Demirjian stages E, F, and G.

### Reliability test

Two examiners (AKM and CML) were tested for inter-observer reliability on 20 randomly selected radiographic images using the 4 methods of recording root development, which gave Cohen’s kappa values of 0.90 to 0.96. During the main survey, duplicate recording was done on (*n* = 50) randomly selected radiographs to test the intra-examiner variability after 1 month of the initial recording. The Cohen’s kappa values ranged from 0.86 to 0.96 with no evidence of systematic errors (*P* > 0.05).

### Data analysis

The data were entered into the computer using EpiData version 4.2.0.0 and double-checked for errors and completeness. The data were then exported to and analyzed using STATA version 13. Numerical values were used to replace the standard designations (D-H and *R*_i_-*A*_c_) for all the methods so as to simplify statistical analysis. Descriptive statistics were used to summarize the data. The difference in root development between left and right mandibular molars was assessed. Furthermore, for the left mandibular third molar, the mean age of tooth development, standard deviation, minimum and maximum values, lower quartile, median, and upper quartile were determined per stage. Student’s *t*-test was used to test differences in dental age based on sex.

### Ethical considerations

The research protocol was approved by the School of Health Sciences Research and Ethics Committee (REF: 2017-040) and the Uganda National Council of Science and Technology (HS 2268). Permission to carry out the study was obtained from the administration of Mulago Hospital. Informed consent was obtained from the adult participants and parents/guardians of children aged 10–17 years. Assent was also obtained from the children less than 18 years in accordance with Helsinki Declaration.

## Results

### Characteristics of the study participants

The study recruited 1089 participants over the 1-year period whose OPGs were considered for the study. However, 31 of the images were excluded because of poor quality, leaving a total of 1058 OPGs. The participants were from the four regions of Uganda, with the majority (*n* = 539, 50.9%) from the central region, followed by the western region (*n* = 135, 12.7%). About 52.6% (*n* = 557) of the participants were females (Table 2). Based on radiographic images, about 37.7% (*n* = 399) of the mandibular third molars were still undergoing crown development (stages A–C, according to Demirjian et al. (1973) method) and were thus eliminated from further analysis leaving 671 images of which females constituted: *n* = 326 (48.6%).

**Table 2:** Frequency distribution of the study participants based on chronological age and sex (*n* = 671)

Age (years)	Females, ***n*** (%)	Males, ***n*** (%)	All, ***n*** (%)
10	6 (100.0)	0 (0.0)	6 (0.9)
11	10 (76.9)	3 (23.1)	13 (1.9)
12	20 (64.5)	11 (35.5)	31 (4.6)
13	25 (55.6)	20 (44.4)	45 (6.7)
14	30 (53.6)	26 (46.4)	56 (8.3)
15	45 (63.4)	26 (36.6)	71 (10.6)
16	35 (55.6)	28 (44.4)	63 (9.4)
17	35 (50.7)	34 (49.3)	69 (10.3)
18	42 (42.9)	56 (57.1)	98 (14.6)
19	29 (35.4)	53 (64.6)	82 (12.2)
20	25 (36.2)	44 (63.8)	69 (10.3)
21	10 (27.0)	27 (73.0)	37 (5.5)
22	14 (45.2)	17 (54.8)	31 (4.6)
**Total**	**326 (48.6)**	**345 (51.4)**	**671 (100)**

The age range of participants was 10–22 years; *n (%)* constitute the number of participants and their percentage

### Left-right mandibular symmetry

For the homologous pairs, the left-right symmetry according to the mean age of root development was quite high among the molars. There was no significant difference between left and right mandibular third molar teeth in the mean age of root development for all the stages (*P <* 0.05, Table 3). The highest mean difference between the right and left mandibular molars at all root stages was 0.1 year.

**Table 3:** Age distribution of participants with teeth at different stages of root development for right and left mandibular molars (*n*=1342)

**DemirjianStages**	**Tooth type**	**No. of teeth**	**Mean age**	**SD**	**Mean difference**	*t*	*P*-value
D	38 48	62 60	13.0 12.9	1.7 1.7	0.1	0.321	0.748
E	38 48	204 205	15.2 15.1	2.1 2.1	0.1	0.285	0.775
F	38 48	165 170	16.8 16.9	2.0 2.0	-0.1	-0.284	0.776
G	38 48	125 125	18.9 18.9	1.7 1.7	0.0	0.190	0.849
H	38 48	115 111	20.0 20.0	1.5 1.6	0.0	0.170	0.865

Root stages D–H are the 5 stages of Demirjian’s classification, 38 lower left mandibular molar, 48 lower left mandibular molar

### Mean age at attainment of stages of third molar root formation using Demirjian et al. (1973) and Solari and Abramovitch (2001) methods

Third molar root development occurred significantly earlier among females compared to males at crown completion (stages D), root initiation (stages E), and root maturation (stage F) stages by both Demirjian et al. (1973) and Solari and Abramovitch (2001) methods (*P* < 0*.*05, Table 4). The mean age for root development among females was different from males by 0.9 year for stages D and E and 1.0 year for stage F Demirjian et al. (1973), while based on Solari and Abramovitch (2001) method, it registered differences of 0.9 year for stage D; 1.0 years, stage E; and 1.4 years, stage F. The mean age at root apex closure (stage H) was 19.8 years for females compared to 20.1 years for males for both methods (Table 4).

**Table 4:** Age distribution of participants according to Demirjian and Solari root development stage of tooth 38 (*n* = 671)

Tooth	Males						Females						
	Formation stages	No. of cases	Mean	SD	95% ***CI***	Medians	No. of cases	Mean	SD	95% ***CI***	Medians	§Mean difference	^**ꝩ**^***p***-value
DGT	D	26	13.5	0.34	12.86–14.18	13.5	36	12.6	0.27	12.07–13.15	12.7	−0.9	0.041
	E	97	15.7	0.21	15.29–16.09	15.7	107	14.7	0.19	14.33–15.08	14.7	−0.9	0.002
	F	82	17.4	0.23	16.91–17.81	17.4	83	16.3	0.21	15.92–16.77	16.3	−1.0	0.002
	G	67	19.0	0.20	18.63–19.40	19.0	58	18.8	0.23	18.32–19.24	18.8	−0.3	0.310
	H	73	20.1	0.16	19.80–20.43	20.1	42	19.8	0.28	19.28–20.39	19.8	−0.9	0.355
Solari	D	26	13.5	0.34	12.86–14.22	13.5	36	12.6	0.27	12.07–13.15	12.7	−0.9	0.041
	E	97	15.7	0.21	15.29–16.12	15.7	107	14.7	0.19	14.33–15.08	14.7	−1.0	0.001
	F	39	17.0	0.28	16.48–17.57	17.0	40	15.7	0.27	15.21–16.27	15.7	−1.4	0.001
	F_1_	43	17.6	0.36	16.88–18.32	17.6	43	16.9	0.29	16.33–17.50	16.9	−0.8	0.093
	G	47	19.0	0.23	18.59–19.49	19.0	49	18.9	0.25	18.38–19.37	18.9	−0.2	0.461
	G_1_	20	19.0	0.43	18.18–19.85	19.0	09	18.2	0.61	17.00–19.42	18.2	−0.5	0.365
	H	73	20.1	0.17	19.76–20.43	20.1	42	19.8	0.28	19.29–20.42	19.8	−0.3	0.354

Root stages D–H are the 5 stages of Demirjian’s classification, while the Solari’s classification has 7stages including two extra root stages: F_1_ and G_1_

### Mean age at attainment of stages of third molar root formation using Moorrees et al. (1963) and Haavikko (1970) methods

Based on Moorrees et al. (1963) and Haavikko (1970) methods, the third molar root significantly developed earlier among females compared to males at root initiation (stage R_i_), quarter root (stage R_1/4_), and half root (stage R_1/2_) stages (*P* < 0*.*05, Table 5). The mean age for root development among females was different from males by 0.9 year for stages R_i_; 1.4 years, R_1/4_; and 1.4 years for stage R_1/2_, using Moorrees et al. (1963) method, while using Haavikko (1970) registered differences of 0.9 year for stage R_i_, 1.0 year stage R_1/4_, and 1.4 years for stage R_1/2_. The mean age at root apical closure was 19.8 years for females compared to 20.1 years for males for both methods (Table 5).

**Table 5:** Age distribution of participants according to Moorrees and Haavikko root development stages of tooth 38 (*n* = 671)

Method	Males							Females						
	Tooth formation stages		No. of cases	Mean	SD	95% ***CI***	Medians	No. of cases	Mean	SD	95% ***CI***	Medians	§Mean difference	^**ꝩ**^***p***-value
MFH	D	R_i_	26	13.5	0.36	12.83–14.2	13.5	36	12.6	0.28	12.08–13.18	12.6	−0.9	0.029
	E	R_cl_	51	15.1	0.29	14.53–15.68	15.1	52	14.5	0.30	13.85–15.07	14.5	−0.6	0.111
		R_1/4_	46	16.3	0.29	15.78–16.92	16.3	55	14.9	0.26	14.48–15.48	14.9	−1.4	0.001
	F	R_1/2_	39	17.1	0.28	16.48–17.63	17.0	40	15.7	0.27	15.19–16.24	15.7	−1.4	0.001
		R_3/4_	43	17.6	0.34	16.90–18.30	17.6	43	16.9	0.31	16.31–17.56	16.9	−0.7	0.110
	G	R_c_	47	19.1	0.24	18.55–19.51	19.0	49	18.9	0.27	18.34–19.41	18.9	−0.3	0.351
		A_1/2_	20	19.0	0.43	18.16–19.19	19.90	9	18.2	0.59	17.00–19.43	18.2	−0.4	0.458
	H	A_c_	73	20.1	0.16	19.79–20.44	20.1	42	19.8	0.28	19.31–20.42	19.8	−0.9	0.330
Haavikko	D	R_i_	26	13.5	0.35	12.86–14.22	13.5	36	12.6	0.29	12.09–13.21	12.6	−0.9	0.029
	E	R_1/4_	97	15.7	0.21	15.25–16.11	15.7	107	14.7	0.19	14.33–15.11	14.7	−1.0	0.001
	F	R_1/2_	39	17.1	0.28	16.53–17.61	17.0	40	15.7	0.26	15.21–16.23	15.7	−1.4	0.000
		R_3/4_	43	17.6	0.36	16.88–18.31	17.6	43	16.9	0.28	16.38–17.48	16.9	−0.7	0.110
	G	R_c_	67	19.1	0.20	18.66–19.42	19.0	58	18.8	0.23	18.31–19.23	18.8	−0.2	0.405
	H	A_c_	73	20.1	0.16	19.78–20.45	20.1	42	19.8	0.28	19.29–20.42	19.8	−0.3	0.288

Root stages R_i_–A_c_ are the 8 stages of MFH’s classification, while the Haavikko’s classification has only 6 root stages excluding MFH root stages R_cl_ and A_1/2_

## Discussion

The present study evaluated third molar development in Ugandan adolescents and young adults resident in the cosmopolitan city of Kampala, which is the capital city of Uganda. Kampala is a commercial hub and has a day population of 3.5 million people with a well representation of all tribes in Uganda. The study population represented urban Ugandans who live on both refined and non-refined (fibrous) diets. The findings in the present study showed no big differences in timing of the third molar root development and other Black populations except South African Blacks who tended to present with higher ages when the root development is at half-length and apex half-closed stages. These differences could have been due to varying study methods or the cosmopolitan nature of the South African population. On the whole, however, studies on dental development in sub-Saharan populations are too few to give a good comparison.

On the other hand, this study found notable differences with other ethnic groups, especially among White British, White Americans, Germans, and the Japanese. The differences ranged from a few months to 2 years earlier throughout the third molar root development stages among Ugandans. Generally, these findings were in agreement with other studies on third molar development among Blacks taking place earlier than other populations. The observed racial differences have been associated with palatal dimensions, where larger dimensions have been observed in Africans compared to other races.

### Age estimation based on third molar root development

Third molar root development is considered an important biological marker for growth during the late adolescence because at this time, other markers would have achieved adult morphology. For instance, in the 15–18-year age group, the ossification of the hand-wrist bones is unreliable marker because these bones would have matured and their epiphyses fused (Schmeling A, 2006). Similarly, the onset of secondary sexual characteristics would have occurred at this time (Rosenfield, Lipton, & Drum, 2009). This makes the third molar an ideal marker for age estimation in late adolescence; therefore, this study sets out to establish age estimates based on distal root development of the mandibular third molar tooth.

### Homologous pairs (left-right) of third mandibular molar

The left-right symmetry in root development for the mandibular third molars was very high in this present study (Table 3). There were no significant differences in the mean age of root development observed in all the stages of homologous pairs of third molars in either males or females (*P ≥* 0.05). The highest mean difference between the left and right mandibular third molars for all root stages was 0.1 year at stages D, E, and F (Table 3). Since these differences were not statistically significant and were not consistent throughout the root stages, moreover, neither side was considered more advanced compared to the other; it follows that any of the mandibular third molar could be used in age assessment. In the present study, the left mandibular third molar (tooth 38) was used to establish age estimation in the Ugandan population. These findings have been reported among Americans (Mincer, Harris & Berryman, 1993), Austrians (Meinl, Tangl, Huber, Maurer & Watzek, 2007), and Black Africans (Olze et al., 2006). Meinl et al. (2007) proposed that in instances when asymmetry is observed, the average dental age of the left and right molars could be considered.

### Sex differences in mandibular third molar root development

In the present study, there were sex differences in root development where females had development of root stages earlier compared to males (Tables 4 and 5). The mean age differences between the females and males ranged between 0.9 and 1.4 years for all the root development stages. These findings are in support of reports from other African populations (Olze et al., 2006). However, the sex difference was not significant, which is in contrast to other studies among Arabs and Africans of Sudanese origin (Elamin, Hector, & Liversidge, 2017), Botswanese (Cavrić, Vodanović, Marušić, & Galić, 2016), Turkish (Sisman et al., 2007), and Brazilians (de Oliveira, Capelozza, Lauris, & de Bullen, 2012), although exceptional traits have been observed in many other populations, where third molar development occurs earlier among the males compared to females for American Whites (Mincer et al., 1993), French Canadians (Levesque et al., 1981), Indians (Kanmani, Srinivasan, & Daniel, 2012), Bangladeshi (H. M. Liversidge, 2008), Turkish (Sisman, Uysal, Yagmur, & Ramoglu, 2007), Austrians (Meinl et al., 2007), and Spanish (Prieto, Barbería, Ortega, & Magaña, 2005).

### Mean age at end of crown mineralization/root initiation

The age of end of crown mineralization (based on Demirjian stage D) and root initiation (based on MFH and Haavikko stage R_i_) was found at 12.6 (95% *CI*, 12.07–13.15) years for females and a few months later at 13.5 (95% *CI*, 12.86–14.18) years for males (Tables 4 and 5).

These findings for the Ugandan females are corroborated by earlier studies done among Ugandans (Mwesigwa et al., 2019) and Botswanese (Cavrić et al., 2016) where stage D was found at 12.6 and 12.4 years, respectively. Similarly published tables from Moorrees’ original work by Harris and Buck (Harris & Buck, 2002) showed stage R_i_ was within the same age range at 12.9 years for North American White children. Similarly, various other populations like South African Blacks (13.6 years) (Olze et al., 2006), Turkish (13.6 years) (Sisman et al., 2007), and sub-Saharan Africans (13.7 years) (H. M. Liversidge et al., 2017) stage D were just a few months later in comparison with the present Ugandan population, while Africans of Sudanese origin (14.2 years) (Elamin et al., 2017), Indians (14.8 years) (H. M. Liversidge et al., 2017), Japanese (14.7 years) (Arany, Iino, & Yoshioka, 2004), Austrians (15.4 years) (Meinl et al., 2007), Spanish (15.1 years) (Prieto et al., 2005), and UK Whites (15.0 years) (H. M. Liversidge et al., 2017) were more than a year late.

Likewise, Ugandan males also displayed results similar to other Black populations including Botswanese at 12.6 years (26), South African Blacks (13.4 years) (Olze et al., 2006), and sub-Saharan Africans (14.1 years) (H. M. Liversidge et al., 2017), although among others like Black Africans from Sudan (14.5 years) (Elamin et al., 2017) and American Blacks (14.4 years) (Blankenship, Mincer, Anderson, Woods, & Burton, 2007) there were a few months difference observed. Other populations including the Turkish (Sisman et al., 2007) and Brazilians (de Oliveira et al., 2012) at 12.9 years, Iranians at 13.4 years (Jafari et al., 2012), and Indians (14.1 years) (H. M. Liversidge et al., 2017) were also within the same age range as males in this present study. Various other populations including Arabs (14.7 years) from Sudan (Elamin et al., 2017), Japanese (14.8 years) (Arany et al., 2004), Spanish (15.0 years) (Prieto et al., 2005), and White children from the UK (14.7 years) (H. M. Liversidge et al., 2017) were a few months later in comparison with the Ugandan population. Although American Whites (15.7 years) (Blankenship et al., 2007), Austrians (16.1 years) (Meinl et al., 2007), Germans (16.3 years), and Japanese (18.2 years) (Olze et al., 2004) attained this stage at remarkably much more advanced ages, this variation could be due to racial differences.

### Mean age when root is half-way developed

The root at half-length (stage F) based on Demirjian et al. (1973) occurred at 16.3 years for females (Tables 4 and 5), which is comparable to 16.8 years in Botswanese (Cavrić et al., 2016), 16.1 years in Black Americans (Blankenship et al., 2007), 16.6 years in Brazilians (de Oliveira et al., 2012), and 16.8 years in Spanish (Prieto et al., 2005). However, the Ugandan females had their root developed half way earlier than South African Blacks (Olze et al., 2006), White Americans (Garn, Lewis, & Bonné, 1962b), and Iranians (Jafari et al., 2012).

The Ugandan males had their root half way developed at 17.4 years, which corroborates findings in Japanese (Arany et al., 2004) and American Whites (Mincer et al., 1993). Additionally Ohio-born White Americans were 16.7 years (Garn et al., 1962a); Iranians, 16.8 years (Jafari et al., 2012); Botswanese, 16.6 years (Cavrić et al., 2016); American Blacks, 16.6 years; (Blankenship et al., 2007); Brazilians, 15.9 years (de Oliveira et al., 2012); and the Spanish, 16.4 years (Prieto et al., 2005). However, higher values were reported among South African Blacks (18.6 years) (Olze et al., 2006) and Japanese (20.4 years) (Olze et al., 2003).

Based on Solari and Abramovitch (2001) and Haavikko (1970) methods, the root development stages (R_1/2_ & R_3/4_) were attained at 15.7 and 16.9 years for females and at 17.0 and 17.6 years for males, respectively. When compared with other reports (Harris & Buck, 2002), females reached this root development stage at a comparable age: 15.8 and 16.4 years, respectively. However, in comparison with Moorrees’ original data (Harris & Buck, 2002), Ugandan males appeared to develop these stages close to 2 years later.

### Mean age when root development is at complete length

The root development at complete length when the apex is half closed (Demirjian et al., 1973, Haavikko, 1970) occurred at 18.8 years for females and 19.0 years for males (Tables 4 and 5). The findings for Ugandan females are in support of other reports among Ugandans (Mwesigwa et al. (2019), the Finnish (Haavikko (1970), Brazilians (de Oliveira et al., 2012), and Botswanese (Cavrić et al., 2016). However, the same root development stages in the present study (Tables 4 and 5) occurred slightly earlier when compared with Austrians (Meinl et al., 2007) and Chinese (Zeng, Wu, & Cui, 2010).

On the other hand, the findings for the Ugandan males corroborated those among the Chinese (Zeng et al., 2010), Botswanese (Cavrić et al., 2016), Japanese (Arany et al., 2004), Iranians (Jafari et al., 2012), and Franco Canadians (Levesque et al., 1981). This was in contrast to 1–2 years earlier in comparison with other reports among Austrians (Meinl et al., 2007) and South African Blacks (Olze et al., 2006).

Based on Solari and Abramovitch (2001) and Haavikko (1970) methods, root development at complete length when the apex is half closed occurred at 18.9 and 18.2 years for females and 19.1 and 19.0 years for males (Tables 4 and 5). The findings for Ugandan females were comparable to Indian population (Liversidge et al., 2017), Africans of Sudanese origin (Elamin et al., 2017), and sub-Saharan Africans, although in contrast to British and Malaysians (Liversidge et al., 2017), which occurred a few months later than in the present study. Similarly, the findings for the Ugandan males corroborated reports among sub-Saharan Africans Indians (Liversidge et al., 2017), Africans of Sudanese origin (Elamin et al., 2017), and British and Malaysians (Liversidge et al., 2017), although much lower ages are reported by Moorrees et al. (1963).

### Mean age when root development is at complete apex closure

In the present study, the mean age at apex closure (stages H and A_c_) was at 19.8 for females and 20.1 years for males (Tables 4 and 5). These findings are comparable to other studies among Ugandans (Mwesigwa et al., 2019). Similar findings were also reported for males by Moorrees and co-workers (1963) and Nystrom and co-workers (2007). Despite the relatively similar values at root development stage H, the findings should be taken with caution because of the associated challenges in determining the end point of this stage (Roberts, McDonald, Andiappan, & Lucas, 2015). This is because tooth development is boundless at its upper limit, for instance different studies have enrolled participants with varying upper age limits ranging from 21 years, 22 years (Mwesigwa et al., 2019), 23 years (Cavrić et al., 2016), 24.9 years (Blankenship et al., 2007), and 25 to 26 years (Olze et al., 2004). Inadvertently, this affects the mean age for this stage, and furthermore, some teeth with open apices may be missed depending on the upper age limit set for the study. Nonetheless, in order to overcome these challenges, the present study used data from a previous Ugandan study (Mwesigwa et al., 2019) to set the upper age limit to 22 years. This was decided because in the previous study by 22 years, all the third molars had undergone complete tooth development and thus had their apices closed. Other studies excluded this stage arguing that once the root is mature, age cannot be estimated from development (Liversidge et al., 2017).

### Limitations of the study

Radiographic identification of root development stages is subjective especially at stages G_1_ and A_1/2_ denoting apex half closed based on modified Demirjian method (Solari & Abramovitch (2001) and Moorrees et al. (1963). However, in order to minimize error, the two investigators (AKM and CML) trained and calibrated on criteria for recording root development before the study. Nevertheless, these challenges are not limited to the present study but have been reported by other workers (De Salvia A, Calzetta C, Orrico M, & De Leo D, 2004, Dhanjal et al., 2006). Furthermore, due to the different classifications of the root development stages employed in the various methods in the present study, the findings should be interpreted with caution.

## Conclusions

The likely age for crown completion (Demirjian stage D)/root initiation (Moorrees stage R_i_) for this population was found at 12.6 years for females and 13.5 years for males, while complete apex closure was at 19.8 for females and 20.1 for males.

On the other hand, the likely age at the in-between stages when the root development is at half length (stages F & F_1_ and R_1/2_ & R_3/4_) ranged between 17.0–17.6 years for males and 15.7–16.9 years for females.

Generally, the present study showed existence of small differences ranging from a few months to 2 years in third molar root development between different ethnic groups in both males and females. The findings of the present study provide age reference standards based on third molar root development specific to the Ugandan adolescent and young adult population. They can be used to formulate baseline standards and utilized as reference materials to assess third molar maturity for forensic purposes. However, further studies are needed in this area of third molar development in other African groups, especially Ugandans to create data bases for comparison.

## Data Availability

The datasets used and analyzed during the current study are available from the corresponding author on reasonable request.

## Abbreviations

OPGs: Orthopantomograms
DGT: Demirjian, Goldstein, and Tanner
MFH: Moorrees, Fanning, and Hunt method
SD: Standard deviation
AKM: Annet Kutesa Mutebi
CML: Catherine Lutalo Mwesigwa
